# Sucrose Concentration and Fermentation Temperature Impact the Sensory Characteristics and Liking of Kombucha

**DOI:** 10.3390/foods12163116

**Published:** 2023-08-19

**Authors:** Gil Cohen, David A. Sela, Alissa A. Nolden

**Affiliations:** 1 Department of Food Science, University of Massachusetts Amherst, Amherst, MA 01003, USA; 2 Department of Nutrition, University of Massachusetts Amherst, Amherst, MA 01003, USA; 3 Department of Microbiology and Physiological Systems, University of Massachusetts Medical School, Worcester, MA 01655, USA

**Keywords:** fermentation, titratable acidity, fermented tea, sensory analysis, physical analysis

## Abstract

Kombucha is a fermented tea beverage consumed for its probiotics and functional properties. It has a unique sensory profile driven by the properties of tea polyphenols and fermentation products, including organic acids. Fermentation temperature and sucrose content affect the fermentation process and the production of organic acids; yet less is known about their impacts on the sensory profile and consumer acceptance. Thus, we aimed to examine the impact of sucrose concentration and fermentation temperature on sensory attributes and liking. For this study, kombucha tea was fermented at three different concentrations of sucrose and fermented at two temperatures for 11 days. Fermentation was monitored by pH, brix, and titratable acidity, and consumers (*n* = 111) evaluated the kombucha for sensory attributes and overall liking. The fermentation temperature resulted in significant differences in titratable acidity, with higher temperatures producing more organic acids, resulting in higher astringency, and suppressed sweetness. The lower fermentation was reported as significantly more liked, with no difference in liking between the 7.5% and 10% sucrose kombucha samples. Fermentation temperature had the greatest impact on the sensory profile rather than sucrose concentration, which had a greater effect on the fermentation rate and production organic acids.

## 1. Introduction

Kombucha is a fermented tea beverage that has been consumed for centuries, dating back to 220 BCE, and was introduced from China to Eastern Europe [1,2]. While this beverage has been consumed for many years, it has recently garnered renewed commercial success around the world for its probiotic benefits [2,3,4]. The kombucha market is growing, with the current U.S. market valued at approximately 2.64 billion $USD as of 2021, and it is expected to reach 9.7 billion $USD by 2030 [3]. This is driven, in part, by its perceived nutraceutical properties such as its antioxidant activity, aiding digestion, and lowering cholesterol [4].

The beverage is made by fermenting black or other types of tea with sucrose and using previously fermented tea as the starter culture. It is generally prepared using sweetened black tea (*Camellia sinensis*); however, other types of tea, such as green or oolong, can be used. The starter tea is referred to as the “mother” symbiotic culture of bacteria and yeast (SCOBY), which initiates fermentation [5,6,7]. According to the prior literature, most kombuchas are made with 10% *w*/*v* sucrose concentration and fermentation is held at temperatures ranging from 18 to 30 °C [2,8,9,10]. Fermentation is carried out for 7 to 14 days [11]. However, some studies have exceeded 14 days to observe the bacterial and chemical dynamics [8,12]. The microorganisms that dominate this probiotic tea are mostly acetic acid bacteria, yeasts, and lactic acid bacteria. The dominant yeast and bacterial genera are *Zygosaccharomyces* and *Komagataeibacter*, respectively [13]. The SCOBY metabolizes sucrose and ethanol to produce organic acids such as acetic, glucuronic, and gluconic acids that provide the beverage’s unique sensorial attributes, with acetic acid providing the dominant acid [11,14,15]. The pH and titratable acidity also play a role in the kombucha’s sensory attributes. The tartness and sourness of kombucha have been associated with the pH, titratable acidity [14], and volatile organic compounds [16].

While kombucha has become an alternative to soft drinks, it can contain between 1 and 24 g of sugar per serving of kombucha [5]. Serving sizes vary across products from 8–16 oz. (250–480 mL); therefore, the present study considers 250 mL a standard serving size. In comparison, soft drinks can contain 26 g or more of sugar per serving (250 mL). While kombucha may provide less sucrose per serving than soft drinks, drinking a serving of kombucha with 24 g of added sugar is roughly half the recommended daily value for added sugar intake (based on a 2000-calorie diet). Therefore, it is important to identify how different concentrations of sucrose impact the sensory attributes and determine the lowest concertation of sucrose that can be used without compromising consumer liking.

There is extensive prior research on the production of kombucha and fermentation characteristics such as substrate concentration, type of substrate, tea type, and fermentation temperatures [1,7,10,12,17]. Much of this work has provided valuable information on the impact of fermentation temperature on bacterial, physical, and fermentation characteristics. There is a scientific gap, however, in understanding the impact that these conditions have on the sensorial properties of kombucha [5]. One study discussed the resulting microbial loads following fermentation at 20 °C and 30 °C, noting a difference in the production of gluconic and glucuronic acids [12]. However, the most abundant acid in kombucha, acetic acid, was not quantified and did not undergo sensory analysis. One of the most comprehensive studies examining the sensory profile of kombucha examined products fermented at two concentrations of sucrose (63 g/L and 94 g/L; equivalent to roughly 16 and 24 g per serving (250 mL), respectfully) and fermented at two temperatures (21 °C and 25.5 °C) [18]. The study concluded that fermentation temperature and sucrose concentration significantly impacted the sensory profile, with lower temperature and higher sucrose concentrations producing a higher sweetness intensity [18]. The quantitative descriptive sensory analysis results provide a comprehensive assessment of the sensory profile, but it does not directly assess consumer liking. In the study by Phetxumphou and colleagues (2023), the lowest sucrose concentration was 6.3% (*w*/*v*) or roughly 16 g of added sugar per serving (250 mL). The study reported herein expands on this finding by investigating a wider range of sucrose concentrations (5%, 7.5% and 10%), which would equate to 12.5 to 25 g of sucrose per serving, helping to determine if lower amounts of sucrose can result in a well-liked kombucha beverage.

Due to recommendations to reduce added sugar intake, it is important to determine if kombucha can be fermented at lower concentrations of sucrose and how this impacts flavor attributes and consumer liking. Therefore, the aim of the present study is to examine the effect of sucrose concentration and fermentation temperatures on the perceived intensity of sensory attributes and overall liking. This will inform the judicious selection of the lowest sucrose concentrations that will not compromise the liking or sensory profile of kombucha tea. Through sensory testing, the effects of sucrose concentration and fermentation temperature on the flavor intensities and liking of kombucha were observed. Chemical and analytical sampling was conducted to follow fermentation patterns and verify the safety of the product. In addition, titratable acidity and pH are crucial factors in the acidity and sourness of a food product [19], thus the effect of titratable acidity and pH on the products were evaluated over the period of fermentation.

## 2. Materials and Methods

### 2.1. Kombucha Preparation

Kombucha tea was prepared in a food-grade facility following good manufacturing practices. Ingredients were sourced from local grocery retailers. Starter culture tea was prepared by fermenting 1 L of black tea, using 8 g/L of loose-leaf black tea (Lipton, India) [11], 100 g of white granulated sugar (Stop and Shop, Quincy, MA, USA), and 10% starter tea from a commercial raw kombucha brand (GT’s Living Foods, Vernon, CA, USA) [4]. The tea was steeped in boiling water for 10 min and then cooled to room temperature before adding the raw kombucha [6]. Once the raw kombucha was mixed in, the starter tea was placed in a 30 °C incubator to ferment for two weeks prior to experimentation. A 40 L batch of black tea was distributed into 12 1-gallon food-grade glass vessels (ULINE, Pleasant Prairie, WI, USA). Glassware was sterilized in boiling water for 10 min before use. After steeping the loose-leaf tea for 15 min [12], the tea leaves were strained using a cheesecloth and dispensed into glass jars. Each vessel of tea contained 3.2 L. Based on previous studies, this experiment fermented kombucha at three concentrations of sucrose 5.0% (50 g/L), 7.5% (75 g/L), and 10% (100 g/L) *w*/*v*% and at two fermentation temperatures, 20 °C (+/− 1.5 °C) and 30 °C (+/− 1.5 °C) [10,12]. Preliminary experiments resulted in variations in small-scale fermentation. To minimize the variability between experimental conditions, the experiment was replicated (i.e., carried out in duplicate) within a single experiment. Duplicate batches for each experimental condition were combined at the end of the fermentation period for sensory testing. After the tea and sucrose mixtures reached room temperature, 224 mL (7% *v*/*v*) of the prepared liquid starter culture tea was mixed into each batch [13]. Fermentation was halted based on pH and titratable acidity measurements on day 11.

### 2.2. Chemical Analysis: pH, Titratable Acidity, Brix

The pH, brix, and titratable acidity measurements were taken daily throughout the fermentation period. Triplicate measurements were taken for each fermentation vessel, resulting in six measures for each experimental condition. Each sample (30 mL) was drawn using a sterile wine thief (E.C. Kraus, Independence, MO, USA) and held in 50 mL falcon tubes (Fisher Scientific, Newington, NH, USA) for analysis. The pH meter (Oakton pH 6+, Vernon Hills, IL, USA) was calibrated daily using pH buffers 4.0, 7.0, and 10.0. The brix refractometer (Milwaukee Instruments MA871, Rocky Mount, NC, USA) was calibrated using distilled water. Sucrose concentrations were measured in units of sucrose (°Brix). Brix measurements from day 0 and day 11 were converted to specific gravity and used to estimate the amount of ethanol in the final product using a simple equation [20]. Manual titrations were performed using 0.1 N NaOH (titrant) and phenolphthalein (color indicator). Titratable acidity was expressed in units of acetic acid. Once fermentation was completed, the cellulosic biofilm was removed from the experimental batches, and teas were stored in a 4 °C food-safe refrigerator. Duplicate batches were combined immediately prior to the sensory evaluation. Alcohol by volume percentages were calculated (following methods described elsewhere [20]). For commercial kombucha, U.S. regulations mandate that the alcohol content be under 1.2%. All batches prepared contained 0.5% or less ethanol, thus considered non-alcoholic. Participants were aware that samples contained trace alcohol in the consent form and the pre-screener questionnaire.

### 2.3. Sensory Analysis

Participants were recruited from the University of Massachusetts and the surrounding area. Individuals were eligible to participate based on the following inclusion criteria: 18 years or older, no tongue, lip, or cheek piercings, not currently pregnant or breastfeeding, no loss of taste or smell function due to COVID-19, had not smoked in the last 30 days, and were willing to consume fermented products with trace amounts of ethanol. In total, 148 participants completed the screener, and 111 people participated in the sensory study. Seven participants were removed from the dataset based on their performance during the training session (described below), resulting in 104 participants (age 27.5 ± 8.7 and 54 female). All protocols were reviewed and approved by the University of Massachusetts Amherst Institutional Review Board for Human Research (IRB #4169).

Participants were invited to complete an in-person sensory test (~20 min). For this study, participants rated the intensity of the samples and overall liking of the kombuchas. General questions about kombucha were asked to obtain information on the regularity of consumption, why one chooses to drink kombucha or consume fermented foods, types of flavors or brands they are familiar with, and their concern with added sugar in food products.

Prior to tasting samples, participants received instructions on the use of the generalized Visual Analog Scale (gVAS) and practiced rating 9 remembered or imagined sensations [21]. The gVAS is a scale used to make intensity ratings, with attributes placed at the ends of the scale: 0 (no sensation of any kind) and 100 (strongest imaginable sensation of any kind). This orientation helps participants practice identifying specific intensities in the context of all sensations, not just taste. Additionally, this practice ensures that participants understand how to use the scale appropriately. For this study, participants rated the brightness of a dimly lit room below the brightness of the sun, with a total of 7 participants removed.

Each participant was served six samples in reusable cups containing 30 mL of kombucha. Samples were presented at room temperature and were blinded with 3-digit codes. The order of samples presented was counterbalanced via a Willams Design. Participants were instructed to taste each sample and drink as much or little as they wanted. Participants first rated eight attributes, selected from a larger set of attributes from [18]. The intensities were sweetness, sourness, astringency, vinegar flavor, fizzy or carbonation, apple juice/cider flavor, lemony/citrus flavor, and yeast flavor. While no formal training was provided, descriptions of each attribute were provided. For example, the sweetness was described as the taste of cotton candy, and astringency was described as a puckering or drying sensation often felt after mouthwash or drinking wine. After reporting the intensity, participants reported their overall liking on a 9-point hedonic scale [14,22]. Participants were instructed to rinse between samples with water (filtered via reverse osmosis). Finally, after tasting all samples, participants were asked to rank most liked to least liked for all six samples. All sensory data were collected using Compusense^®^ (Compusense Inc, Guelph, ON, Canada).

### 2.4. Statistical Analysis

Statistical analysis was conducted to assess the relationship between temperature and sucrose concentrations on reported liking via analysis of variance (ANOVA) and a T-test, respectively. Significant ANOVA models were followed with Tukey’s honest significant difference (HSD) to examine differences between pairs. Separate ANOVA models were conducted to determine the impact of sucrose concentration and fermentation temperature and their interaction effect on each sensory attribute and overall liking. A stepwise regression was conducted to identify the attributes associated with reported overall liking. The relationship between titratable acidity and sensory ratings (astringency and sourness) was examined using linear regression with an adjusted R^2^ value reported. Differences in titratable acidity across fermentation temperatures were examined via Student’s *t*-test. All analyses were conducted using RStudio (version 2023.03.1).

## 3. Results

### 3.1. Determination of pH, Brix, and Titratable Acidity

**Figure 1:**
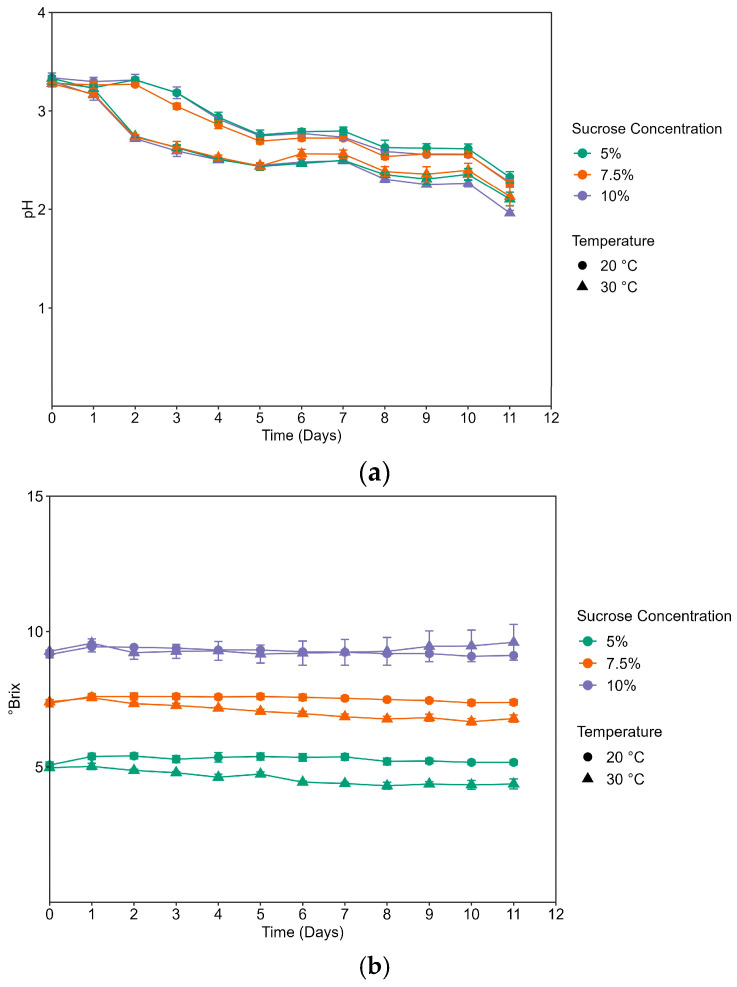

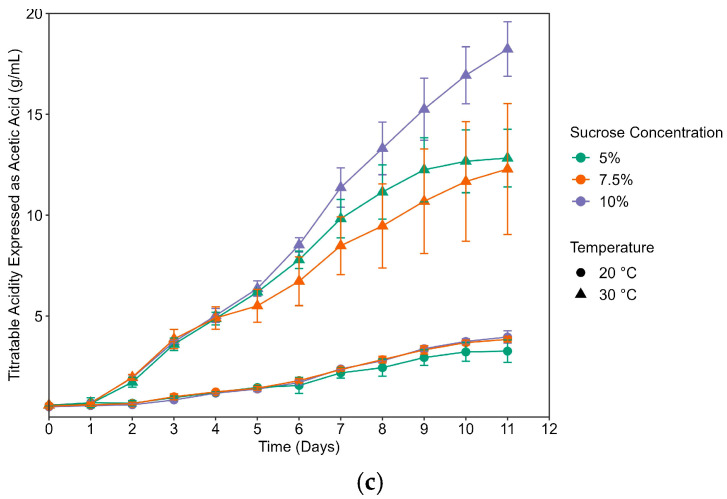
Measurements (mean ± SD) for (**a**) pH, (**b**) Brix, and (**c**) titratable acidity throughout the 11-day fermentation period.

pH, brix, and titratable acidity measurements were recorded daily throughout fermentation (Figure 1). During the fermentation period, all samples decreased in pH and increased in titratable acidity as expected. The pH of kombucha on day 0 (day of preparation) was below 4.6, the pH level at which spoilage organisms are less likely to grow [23]. The final pH of 30 °C batches ranged between 2.25 and 2.38, while 20 °C batches ranged between 1.95 and 2.21 (Figure 1a). Fermentation was halted on day 11, with a final range of pH 1.95–2.38. Sucrose, measured in °Brix, varied over the course of fermentation (Figure 1b). All samples fermented at 30 °C had a lower pH and higher titratable acidity (expressed in g/L of acetic acid). The titratable acidity of the samples fermented at 20 °C ranged from 3.26 to 3.96 g/L, while 30 °C ranged from 12.83 to 18.24 g/L on the final day of fermentation (Figure 1c). Titratable acidity was significantly associated with fermentation temperature (*t*-test; *p* < 0.05).

Batches were refrigerated following the fermentation and combined prior to sensory analysis and the titratable acidity of the combined batches was evaluated. The final titratable acidity for 20 °C was (3.52 ± 0.6, 4.24 ± 0.06, 4.36 ± 0.03) and for 30 °C was (14.57 ± 0.0, 13.81 ± 0.06, 21.14 ± 0.06), for the 5%, 7.5% and 10% sucrose concentrations, respectfully.

### 3.2. Sensory Evaluation

#### 3.2.1. Summary of Participant Characteristics

A total of 111 participants took part in the study. After removing seven participants that did not complete the training properly (see Section 2.3), the final data set included 104 participants, with 50 identifying as male and 54 identifying as female. The pool of participants reported that 41% did not consume fermented tea. However, 90% answered that they consume fermented foods regularly. Participants that reported that they did not eat fermented foods were prompted by another question asking, “For what reason(s) have you not consumed kombucha?”. A total of 60% of these participants reported liking the taste of kombucha; however, they had not consumed it in the last 6 months. Overall, 61% of participants responded that they generally liked kombucha. Reasons for consuming kombucha included its taste, probiotic or health benefits, trendiness, and availability. Those that reported not consuming kombucha regularly mentioned that they did not like the taste, cost, carbonation, had not tried it before, or did not have it readily available. Samples prepared in this study were intentionally not carbonated. Carbonation is considered an oral irritant and can affect the overall liking of a beverage [24]. Since one of the objectives of this study focused on reducing the added sugar content of kombucha, it is useful to understand whether consumers are aware of the added sugar content in commercial kombucha beverages. Participants were asked to identify the amount of added sugar typically found in a serving of kombucha. Based on the market assessment of commercial kombucha beverages, the average amount per serving is 13 g. In the present study, 11% of participants selected 20 g, with 34% correctly selecting the average amount of added sugar (13 g), whereas 55% thought kombucha contained less than 13 g. This suggests that most consumers perceive a typical kombucha product to contain less added sugars. It is important to note, however, that not all participants were regular drinkers of kombucha.

#### 3.2.2. Overall Liking

**Figure 2:**
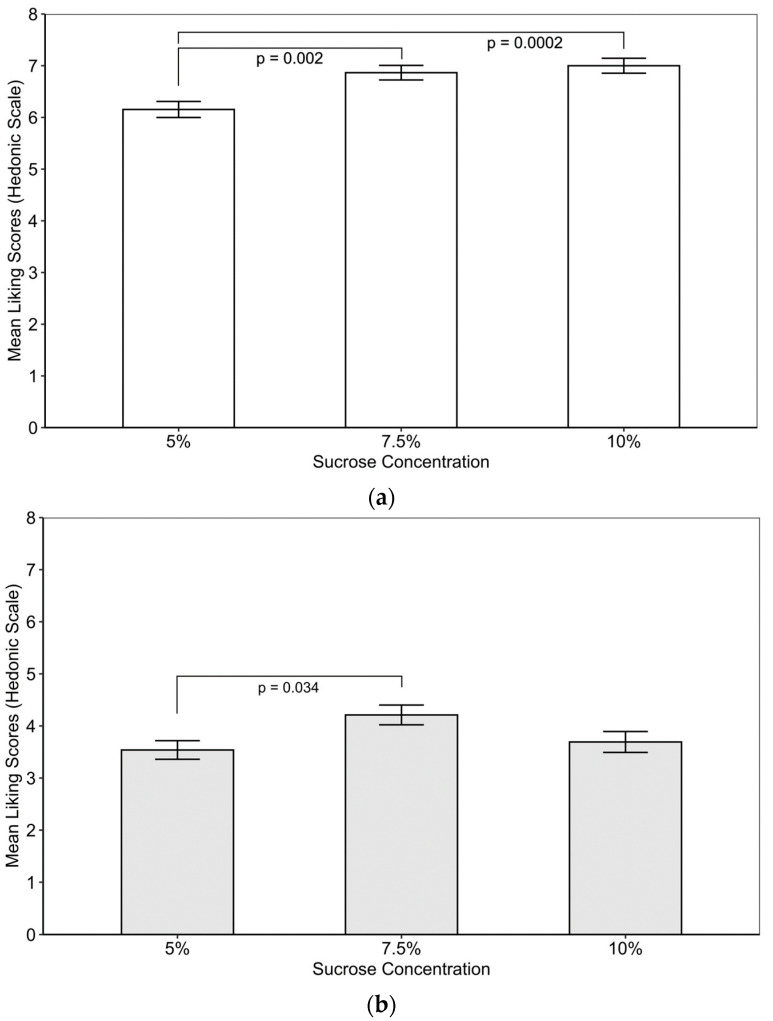
Mean liking scores (±SEM) for kombucha with 5%, 7.5% and 10% (*w*/*v*) fermented at (**a**) 20 °C and (**b**) 30 °C.

The overall liking of the kombucha samples is presented in Figure 2. Separate ANOVA models examined the effect of sucrose concentrations on overall liking for each fermentation temperature. For 20 °C, there was a significant relationship between sucrose and overall liking [F(2, 309) = 9.58; *p* < 0.0001]. The post hoc test revealed differences between 5% and 10%, as well as 5% and 7.5% sucrose concentrations (*p* < 0.05), without other significant differences between the three sucrose levels (Figure 2a). Similarly, there was a significant relationship between sucrose and the overall liking for samples fermented at 30 °C [F(2, 309) = 3.45; *p* = 0.033]. The post hoc test results revealed significant differences between 5% and 7.5% sucrose (Figure 2b). To test whether temperature had a significant relationship with reported liking, *t*-tests were conducted at each sucrose concentration. There was a significant difference at each sucrose concentration (*p* < 0.05), with samples fermented at 20 °C liked significantly more at each sucrose concentration than those fermented at 30 °C. In other words, the greatest variation in liking was observed across fermentation temperatures. All room temperature batches were rated near like slightly to like moderately on the hedonic scale, whereas 30 °C samples were rated from “dislike slightly” to “dislike moderately” (Figure 2).

A repeated-measures ANOVA was conducted to determine the interaction between temperature and sucrose concentration on overall liking. The model revealed no significant interaction effect (*p* = 0.07); however, there was a significant effect of sucrose [F(2, 618) = 8.9; *p* = 0.0002)] and temperature [F(1, 618) = 425.7; *p* < 0.0001].

#### 3.2.3. Sensory Attributes

**Figure 3:**
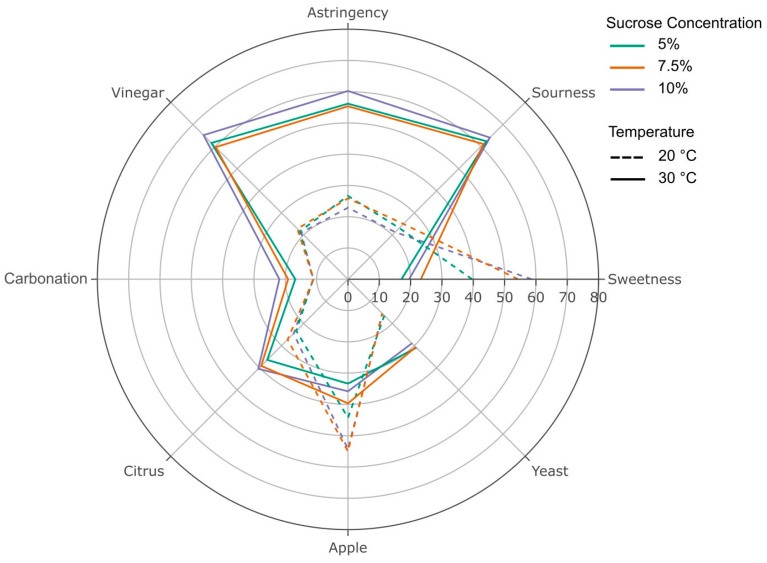
Sensory profile of the kombucha beverages fermented at 20 °C and 30 °C (lines: dashed and solid) with 5%, 7.5%, and 10% sucrose (colors: green, orange, purple). Mean intensity ratings reported on general visual analog scale (gVAS) for eight flavor attributes.

Participants rated the perceived intensity of sweetness, sourness, astringency, vinegar flavor, apple flavor, carbonation, citrus flavor, and yeast aroma or flavor. Mean reported intensities are reported in Figure 3.

A forward stepwise regression was conducted to determine the attributes that are significantly associated with reported overall liking. The model revealed that sweetness, sourness, astringency, vinegar flavor, apple flavor, and yeast flavor were significantly associated with overall liking (Table 1) and that these attributes explained 51.8% of the variability in overall liking. Sweetness intensity had the strongest relationship with overall liking, with the second most influential attribute being astringency, which negatively influenced overall liking. Apple flavor was the only other attribute with a significant positive relationship with overall liking, with sourness, vinegar, and yeast flavor negatively influencing overall liking.

The reported attribute intensities were assessed to be driven by sucrose concentration and fermentation temperatures. Separate ANOVA models were used to identify the effect of sucrose and temperature and their interaction on the reported intensity of each flavor attribute. The effect of sucrose on each of the flavor intensities showed statistical significance (all *p* < 0.0001) for the perception of sweetness and apple flavor ([F(2, 618) = 16.1] and [F(2, 618) = 5.6], respectfully). Temperature, in contrast, was significantly associated with the rated intensity of sourness [F(1, 618) = 451.9], vinegar flavor [F(1, 618) = 433.1], sweetness [F(1, 618) = 310.5], astringency [F(1, 618) = 247.8], yeast flavor [F(1, 618) = 57.9], apple flavor [F(1,618) = 48.4], lemon flavor [F(1, 618) = 47.8], and carbonation [F(1, 618) = 27.5] (all *p* < 0.0001). There was a significant interaction between fermentation temperature and sucrose concentration on the reported sweetness intensity [F(2, 618) = 7.4; *p* = 0.0006], with no other attributes demonstrating a significant interaction.

**Figure 4:**
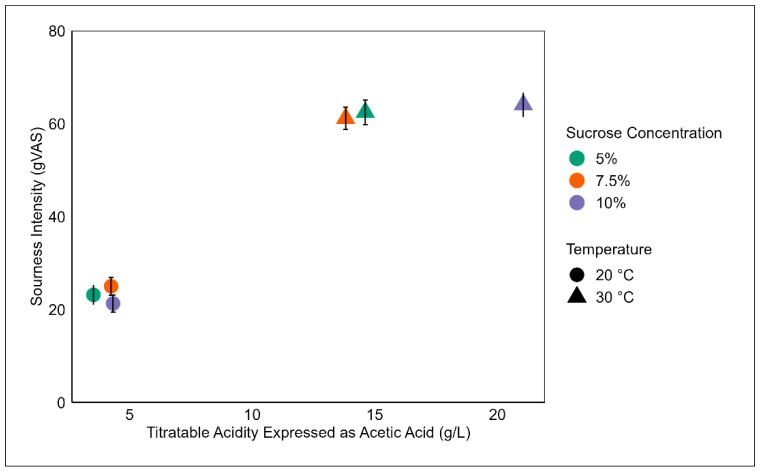
A comparison between mean sourness intensity ratings (±SEM) and total titratable acidity (g/L) for kombucha samples. Intensity ratings were collected using a gVAS, general visual analog scale. Different shapes represent temperature (20 °C, circle and 30 °C, triangle) and different colors represent sucrose concentration (5%, green; 7.5%, orange; and 10%, purple).

Measured titratable acidity was significantly associated with the reported sourness and astringency. A linear model determined that titratable acidity explained 39% of the variability in sourness (Figure 4) and 26.6% of the variability in astringency (*p* < 0.0001). A *t*-test revealed that the reported titratable acidity for samples fermented at 30 °C were significantly higher than samples fermented at 20 °C (*p* < 0.0001).

## 4. Discussion

Fermentation conditions influence the flavor profile [3,5]; yet there is minimal published research on the consumer perception of kombucha [5]. This study extends our understanding of how fermentation temperature and sucrose concentration influence kombucha’s physical and sensory characteristics.

The pH, brix, and titratable acidity in kombucha drive the sensory profile of the fermented tea [5,19,25]. As expected, the decrease in pH coincided with an increase in titratable acidity in all samples. The pH of kombucha generally ranges from 2.5–3.5, with a pH lower than 2.5 indicating the greater microbial depletion of sucrose and the potential for increased ethanol concentrations. Since the pH of the higher-temperature samples, 30 °C, decreased below 2.5, and some below 2.0, the fermentation was halted at 11 days, and alcohol by volume percentages was calculated. A decrease in brix by the end of the fermentation period was not observed, indicating that initial sucrose concentrations exceeded microbial needs in the fermentation process.

A higher fermentation temperature resulted in an increase in qualitative fermentation rates, producing more organic acids (higher titratable acidity), and a faster decline in the pH. The higher-temperature kombucha was liked significantly less than the lower-temperature kombucha samples. The dislike of samples was driven by higher perceived astringency, sourness, and vinegar flavor. This is potentially linked to a significant association between titratable acidity and reported astringency. It is acknowledged that participants may have difficulty differentiating between sensations, specifically, astringency and sour, and possibly vinegar flavor [26]. However, these sensations correspond to a lower pH and higher titratable acidity. Andreson and colleagues (2022) reported a correlation between titratable acidity and perceived sourness for commercial kombucha beverages [25]. Current findings regarding the relationship between the fermentation temperature and sensory profile are supported by a previous study, which concluded that fermentation temperature impacts the sensory profile, noting that a higher fermentation temperature was associated with an increased perceived intensity for sourness, puckering, pungent, astringent, and vinegar flavor [18]. Moreover, this previous report concluded that fermentation temperature explains more variation in the reported intensity of sensory attributes, with sucrose concentration still having a significant, albeit less, impact on the sensory profile. This aligns with the present study, as fermentation temperature and sucrose concentrations independently influence the sensory profile, with the interaction between factors [18].Prior work has assessed the chemical composition of kombucha, including quantifying specific acid content [9,16], antioxidant activity [9,12], and polyphenol content [9,11], which traditionally has been used to predict the flavor profile. However, more empirical evidence is needed to validate this relationship between instrumental measures and consumer’s perceptions for kombucha [27]. Future studies linking chemical analysis with sensory profiles will benefit researchers and the industry.

Perceived astringency is modulated by adding sweet compounds and sweet-related flavors [28,29]. Therefore, one could rationalize that the addition of sucrose would suppress or reduce the perceived astringency and potential sourness. However, prior work demonstrated that the successful suppression of astringency was achieved for lower intensity levels. For the kombucha tested here, participants reported astringency and astringency-related sensations (sour and vinegar) as the dominant sensations reported for the higher-temperature samples, and the addition of sucrose did not appear to be effective at reducing the perceived astringency. Due to higher astringency, sourness, and vinegar sensations negatively influencing overall liking, it is important to identify the ideal pH and titratable acidity to ensure acceptable amounts of these sensations.

The perceived sweetness was the strongest predictor for reported overall liking across all samples. Kombucha fermented at 20 °C produced higher perceived sweetness compared to 30 °C, regardless of sucrose concentration. Sucrose concentrations appeared to drive differences in perceived sweetness and apple flavor but no other attributes, which are less pronounced than studies reporting kombucha’s sensory profile. Phetxumphou et al. (2023) report that lower sucrose concentrations produced beverages with a higher perceived intensity of astringent, yeasty aroma, fizzy, and sour attributes [18]. In addition, authors have reported that sucrose concentrations influenced perceived sweetness and sweet-related attributes (e.g., honey) along with fruity-related flavors (e.g., apple, berry, and grape) [18]. The perceived sweetness was the strongest predictor for reported overall liking across all samples. Kombucha fermented at 20 °C produced higher perceived sweetness compared to 30 °C, regardless of sucrose concentration. Previous studies have reported that sucrose concentrations were important for the perception of taste- and flavor-related sensations. Similar to the present study, Phetxumphou et al. (2023) reported that lower sucrose concentrations produced beverages that had a higher perceived intensity of astringent, yeasty aroma, fizzy, and sour attributes [18]. In addition, authors reported that sucrose concentrations influenced perceived sweetness and sweet-related attributes (e.g., honey) along with fruity-related flavors (e.g., apple, berry, and grape) [18]. One possible reason for this difference is the tea used for fermentation, with the present study examining black tea. As a result, the interaction between sucrose and other tea types or base beverages is likely to result in the formation of different sensory profiles.

The present study examines sucrose concentration and fermentation temperature on the physical and sensory characteristics. These parameters, along with tea type and fermentation time, influence the composition and metabolism of microorganisms [30]. Changes in the microbial community likely drive this effect, as different microbial compositions are associated with physical characteristics and chemical composition, including pH, titratable acid, reducing sugars, polyphenols, acetic acid, ethanol content, and flavor volatiles [31,32,33]. The chemical composition of kombucha, including acid content [9,16], antioxidant activity [9,12], and polyphenol content [9,11], are driven by these fermentation conditions, which drive the final flavor profile [27]. To ensure a stable fermentation, it has been recommended that the initial microorganisms are important to produce desirable characteristics [34,35,36] and that the microbial composition is an important factor contributing to the sensory profile [27]. There is a need for additional studies to examine the interaction between fermentation conditions and microbial community on the physical and sensory characteristics. A greater scientific understanding will help identify the ideal parameters for producing products with a desired sensory profile and health benefits.

Due to recommendations for limiting added sugar and considering consumer preference, the beverage industry should consider reducing the amount of sucrose added. For the sucrose amounts tested in the present study, the lower sucrose concentration (5%) was significantly less than 7.5% and 10% sucrose for the samples fermented at 20 °C. Nonetheless, the lowest sucrose concentration tested was still liked, rated at ‘like slightly’. One potential strategy for increasing perceived sweetness is the addition of sweet-related flavors (e.g., fruit and honey). Additional research is needed to determine if a greater reduction could be achieved on an industrial scale. Based on study findings, there was no difference in liking ratings between 7.5% and 10% (Figure 2a), suggesting a reduced added sugar can be achieved while maintaining liking. While formulations may need to be modified for larger-scale production, upscaling the current formulation, a reduction from 10% to 7.5% sucrose would translate to roughly a 6 g reduction in added sugar per serving (250 mL). The present study highlights the impact of these fermentation parameters on unflavored black tea under controlled conditions at a laboratory scale.

Prior work suggests that the optimal temperature for fermentation is 22–28 °C [10]. While higher temperatures tend to yield faster ferments [37], monitoring the titratable acidity and pH is important, as these parameters appear to drive perceived astringency and sourness, with higher intensities negatively impacting overall liking. While fermentation typically lasts 7 to 14 days, the present study stopped fermentation on day 11, it is possible that if the fermentation had ended earlier, especially in the case of samples fermented at 30 °C, it might have resulted in a lower production of titratable acidity and higher pH levels and higher liking ratings. Identifying the acceptable amount of titratable acidity, driven by fermentation temperature and the ideal sucrose concentration, is important for producing a desirable sensory profile.

## 5. Conclusions

The fermentation temperature and sucrose concentrations impact the development of novel sensorial attributes in kombucha. While both fermentation temperature and sucrose concentration were significantly associated with kombucha’s reported overall liking and sensory profile, temperature had a greater influence than sucrose concentration. Kombucha fermented at a lower temperature received higher liking ratings, driven by the perceived sweetness and apple flavor. The higher fermentation temperature sped up the fermentation rate, producing a higher titratable acidity and lower pH, which was associated with higher astringency and sourness intensity ratings and lower overall liking. While astringency is an authentic sensation of kombucha, higher astringency is likely suppressing the sweetness, which was not overcome by the higher sucrose levels. Results of the present study suggest that 7.5% sucrose provides similar liking of 10% sucrose, but lowering the sucrose to 5% negatively impacted liking. Future studies could examine the possibility of adding sweet-related flavors to improve the overall liking of a lower-sucrose kombucha beverage. In summary, this study highlights the importance of sucrose and fermentation temperature on the development of the sensorial properties of kombucha.

## Figures and Tables

**Table 1:** Summary of stepwise forward regression model. The model determined which variables were significant in overall liking scores. All attributes reported a *p*-value < 0.05.

Stepwise Regression		
**Final Model Summary:**	**Adj. R^2^** **=0.518**	***p*-value** **<0.0001**
**Individual variable contribution**		
Attribute	β coefficient	*p* -value
Sweetness	0.405	<0.0001
Astringency	−0.204	<0.0001
Sourness	−0.163	0.001
Vinegar	−0.111	0.02
Apple	0.084	0.016
Yeast	−0.073	0.023

## Data Availability

Data will be available upon request from the corresponding author.
